# A Prospective Study of the Impact of Transcranial Alternating Current Stimulation on EEG Correlates of Somatosensory Perception

**DOI:** 10.3389/fpsyg.2018.02117

**Published:** 2018-11-20

**Authors:** Danielle D. Sliva, Christopher J. Black, Paul Bowary, Uday Agrawal, Juan F. Santoyo, Noah S. Philip, Benjamin D. Greenberg, Christopher I. Moore, Stephanie R. Jones

**Affiliations:** ^1^Department of Neuroscience, Brown University, Providence, RI, United States; ^2^Department of Biomedical Engineering, School of Engineering, Brown University, Providence, RI, United States; ^3^Department of Psychiatry and Human Behavior, Brown University Medical School, Providence, RI, United States; ^4^Center for Neurorestoration and Neurotechnology, Providence VA Medical Center, Providence, RI, United States; ^5^Butler Hospital, Providence, RI, United States; ^6^Harvard Medical School, Boston, MA, United States

**Keywords:** transcranial alternating current stimulation, somatosensory perception, tactile detection, alpha, neuromodulation

## Abstract

The (8–12 Hz) neocortical alpha rhythm is associated with shifts in attention across sensory systems, and is thought to represent a sensory gating mechanism for the inhibitory control of cortical processing. The present preliminary study sought to explore whether alpha frequency transcranial alternating current stimulation (tACS) could modulate endogenous alpha power in the somatosensory system, and whether the hypothesized modulation would causally impact perception of tactile stimuli at perceptual threshold. We combined electroencephalography (EEG) with simultaneous brief and intermittent tACS applied over primary somatosensory cortex at individuals’ endogenous alpha frequency during a tactile detection task (*n* = 12 for EEG, *n* = 20 for behavior). EEG-measured pre-stimulus alpha power was higher on non-perceived than perceived trials, and analogous perceptual correlates emerged in early components of the tactile evoked response. Further, baseline normalized tactile detection performance was significantly lower during alpha than sham tACS, but the effect did not last into the post-tACS time period. Pre- to post-tACS changes in alpha power were linearly dependent upon baseline state, such that alpha power tended to increase when pre-tACS alpha power was low, and decrease when it was high. However, these observations were comparable in both groups, and not associated with evidence of tACS-induced alpha power modulation. Nevertheless, the tactile stimulus evoked response potential (ERP) revealed a potentially lasting impact of alpha tACS on circuit dynamics. The post-tACS ERP was marked by the emergence of a prominent peak ∼70 ms post-stimulus, which was not discernible post-sham, or in either pre-stimulation condition. Computational neural modeling designed to simulate macroscale EEG signals supported the hypothesis that the emergence of this peak could reflect synaptic plasticity mechanisms induced by tACS. The primary lesson learned in this study, which commanded a small sample size, was that while our experimental paradigm provided some evidence of an influence of tACS on behavior and circuit dynamics, it was not sufficient to induce observable causal effects of tACS on EEG-measured alpha oscillations. We discuss limitations and suggest improvements that may help further delineate a causal influence of tACS on cortical dynamics and perception in future studies.

## Introduction

Since its discovery almost a century ago (Berger, 1969), the alpha rhythm remains one of the most conspicuous yet elusive signals that can be recorded from the human brain non-invasively. Once regarded a “passive idling state,” more recent work has clearly demonstrated that shifts in attention lead to sustained focal changes in alpha activity in visual (e.g., Worden et al., 2000), auditory (e.g., Frey et al., 2014) and somatosensory (e.g., Jones et al., 2010) systems. Though the alpha rhythm likely stems from different generators across sensory systems (Frey et al., 2014), differences in alpha power and/or phase consistently predict sensory perception (Rice and Hagstrom, 1989; Thut, 2006; van Dijk et al., 2008; Jones et al., 2010). This has led to the hypothesis that the alpha rhythm represents a functionally relevant sensory gating mechanism for the inhibitory control of cortical processing (see reviews, Klimesch et al., 2007; Jensen and Mazaheri, 2010).

Nevertheless, the question of whether alpha truly exerts a causal influence on sensory perception, or is a mere correlate, remains a topic of debate. In light of this, recent efforts have focused on using non-invasive brain stimulation (NIBS) to provide evidence that modulation of alpha power and phase can causally impact perception (e.g., Romei et al., 2010). Transcranial alternating current stimulation (tACS) is one such NIBS approach that is considered relatively safe, accessible and inexpensive. In this method, a sinusoidal current of a functionally relevant frequency is applied at the scalp between two stimulating electrodes. It has been suggested that the applied current may interact with and thus enhance ongoing rhythmic activity (Antal et al., 2008). For instance, 10 min of tACS over occipital cortex at participants’ endogenous alpha frequency can significantly increase individual-specific alpha power compared to baseline (Zaehle et al., 2010). Such effects can last for an extended period of time (30 min), and depend on dynamic brain states, such as baseline endogenous alpha power (Neuling et al., 2013).

Helfrich et al. (2014) pioneered a novel artifact removal technique to make the claim that such changes in alpha power are specifically due to an “entrainment” effect; parieto-occipital tACS at 10 Hz synchronized individuals’ endogenous alpha rhythm to the applied frequency, and modulated performance in a visual oddball task in a phase-dependent manner. In contrast, an intermittent tACS paradigm (8 s trains/7,200 cycles) demonstrated that analogous alpha power after-effects could exist in the absence of synchronization. This suggests that an alternative, though not necessarily mutually exclusive or independent, plasticity mechanism may account for the perceptual effects observed in the visual system following 10 Hz tACS (Vossen et al., 2015).

Alpha tACS-induced perceptual changes, and corresponding correlates of power and phase, are not just limited to the visual system. For example, oscillating (10 Hz) transcranial direct current stimulation (tDCS) over auditory cortex had a comparable effect; post-stimulation alpha power was enhanced, and detection thresholds in a subsequent auditory detection task were dependent upon the phase of the applied alpha frequency (Neuling et al., 2012). In the somatosensory domain, tACS at individuals’ endogenous alpha frequency over primary somatosensory cortex (SI) modulated tactile detection, such that post-stimulation detection thresholds varied as a function of alpha phase (Gundlach et al., 2017). However, further evidence of alpha power modulation, or entrainment per se, relating to somatosensory perception has not been reported.

Prior magnetoencephalography (MEG) experiments from our group demonstrated that alpha power in primary somatosensory cortex (SI, source-localized) shifted with cued attention. Participants were instructed to either pay attention to the hand or the foot, and then report detection of a brief tactile stimulus at perceptual threshold (i.e., 50% detection probability) to either location. Post-cue/pre-stimulus alpha power increased in the non-attended, compared to the attended, somatic representation. Moreover, the attentional shift was functionally relevant; high SI alpha power was a strong predictor of decreased tactile detection (Jones et al., 2010). This finding, combined with computational neural modeling detailing the neural origins of SI alpha (Jones et al., 2009), suggest that alpha activity may be actively engaged to diminish relay of signals from thalamus to neocortex in the context of somatosensory perception.

Building from these prior reports, the present preliminary study sought to explore whether alpha frequency tACS could modulate alpha power in the somatosensory system, and if the hypothesized modulation would causally impact perception of tactile stimuli at perceptual threshold. Accordingly, using a novel Open Ephys-EEG system (Black et al., 2017; Siegle et al., 2017), we combined electroencephalography (EEG) with simultaneous intermittent tACS (6 s on and off/∼15 min) applied over SI at individuals’ endogenous alpha frequency during our well-established tactile detection task (Jones et al., 2007, 2010; Sacchet et al., 2015).

Our original goal was to record EEG signals concurrent with electrical stimulation in order to test the hypothesis that alpha frequency tACS causally decreases perception via the induction of cortical alpha oscillations. However, there were many limitations to our design that did not allow us to definitively address this hypothesis, and resulted in a small sample for EEG analysis (n = 12). Nonetheless, in the spirit of this special issue, we describe preliminary results that suggest that intermittent alpha tACS can impact behavioral performance and brain dynamics without a measurable effect on EEG-measured somatosensory alpha power. In addition, we applied computational neural modeling designed to simulate macroscale EEG signals (Jones et al., 2007, 2009; Neymotin et al., 2018) to test the hypothesis that such effects could be consistent with changes in synaptic plasticity (Zaehle et al., 2010; Polanía et al., 2012; Vossen et al., 2015; Lafon et al., 2017). We conclude with a detailed discussion of lessons learned from this study that can be used to inform improved design of future studies aimed at modulating brain rhythms and/or sensory perception using NIBS.

## Materials and Methods

### Tactile Detection Task

Prior to the start of the experiment, a training period familiarized participants with the detection paradigm (see below). Following training, individual detection thresholds (∼50% detection) were determined using the parameter estimation by sequential testing (PEST) procedure (Dai, 1995; Leek, 2001; Jones et al., 2007). This threshold value was used as an initial condition for the tactile stimulus at the beginning of the detection paradigm. As in prior reports (Jones et al., 2007), the intensity of the threshold-level stimulus was decreased slightly (by a change of ∼4.5 μm in piezoelectric deflection) if two consecutive correct responses were made, or increased by the same amount if three consecutive incorrect responses were made, in order to account for slight changes in sensitivity of the finger to touch over time. Correct/incorrect response counts were always subsequently reset to zero. Applying this procedure, detection rates in the current study varied around 50%, between ∼20 and 70% detection.

**FIGURE 1:**
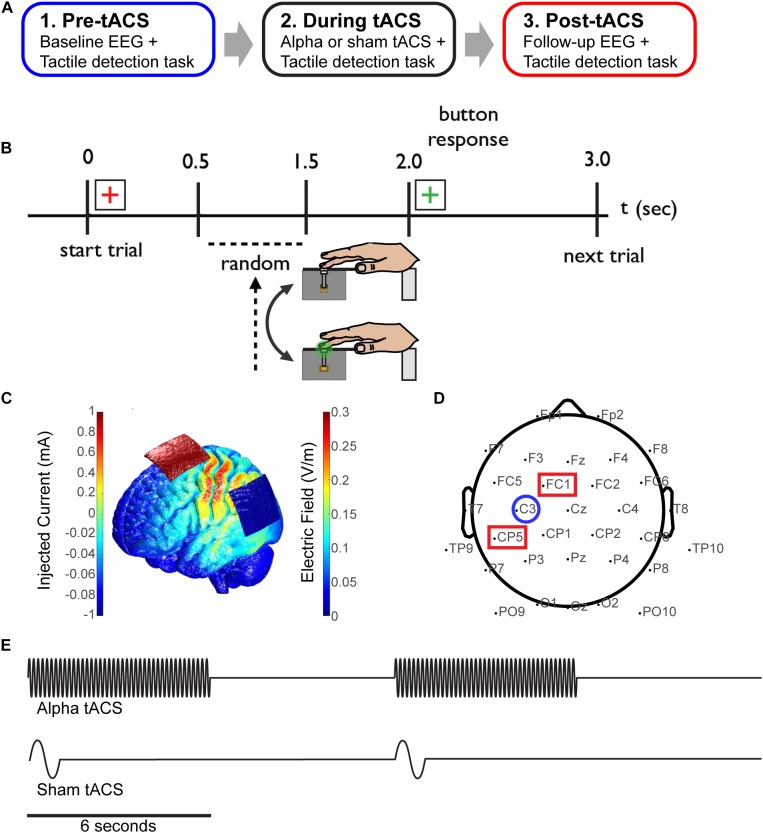
Combining EEG and tACS during a tactile detection task. **(A)** The experimental session was divided into three time blocks; pre-, during and post-tACS (sham or alpha). **(B)** Schematic of the tactile detection task. Participants rested their right hand on a tactile stimulator that delivered brief light taps to the finger (third digit) at perceptual-threshold level, and subsequently reported detection of the stimulus using their left hand. A red cross-hair cued the start of the trial, and a green crosshair cued participants to subsequently select a response. **(C)** Finite element modeling was used to determine a stimulation montage resulting in maximal current flow over somatosensory cortex (see “Materials and Methods”). **(D)** Simultaneous EEG-tACS set-up. Stimulating electrodes were placed over CP5 and FC1 (red squares; International 10–20 system), and data was analyzed from EEG electrode C3 (blue circle), overlying primary somatosensory cortex. **(E)** Schematic of the alpha and sham tACS protocols. Electrical stimulation was applied at participants’ individual alpha frequency at 1 mA for a period of 6 s on/off (alpha tACS, top), or was ramped up to 1 mA at 1 Hz for 1 cycle and then turned off (sham tACS, bottom).

The experimental paradigm was divided into three time blocks; pre-, during and post-tACS (sham or alpha; Figure 1A). During each block, participants were asked to report detection of a tactile stimulus to the third digit (D3) of the right hand (Figure 1B). Within each trial, a red fixation crosshair initially cued the impending stimulus for 2 s. During this time, a threshold-level (as described above, 70% of trials), supra-threshold level (100% detection, 10% of trials) or null tactile stimulus (0% detection, 20% of trials) was presented with a jittered (0.5–1.5 s) delay from the trial start. A green fixation cross subsequently appeared for 1 s, during which time participants provided a response using their left hand. A keypress of ‘1’ indicated that the stimulus was perceived, and ‘2’ indicated that it was not perceived. Participants performed 700 trials in total; 200 trials pre-/post-tACS, and 300 trials during tACS.

Using methods previously reported (Jones et al., 2007), the tactile stimuli consisted of brief light ‘taps’ on the finger from a tactile stimulator, encompassing a plastic screw mounted to a piezoelectric bender (Figure 1B). Stimuli were generated by a 10 ms 100 Hz sine wave using the Psychophysics Toobox extensions (Brainard, 1997; Pelli, 1997; Kleiner et al., 2007) in Matlab, and externally amplified using a PDu100B miniature piezo driver (PiezoDrive).

### Transcranial Alternating Current Stimulation (tACS)

Prior to beginning the experiment, participants were assigned to either the sham or alpha tACS group. Additionally, finite element modeling was performed with the Neuroelectrics Instrument Controller to determine a montage resulting in maximal current flow over electrode C3 (corresponding to the International 10–20 system), overlying somatosensory cortex (Figure 1C). Prior to the task, individual (participant-specific) alpha-band stimulation frequencies were determined by recording 10 s of baseline EEG data (electrode C3; see Figure 1D) while participants were seated and resting quietly with eyes open, and with their hand resting on the tactile stimulator. Peak alpha frequency was calculated within a 7–14 Hz band using a stationary pwelch method. Electrical stimulation was delivered using a neuroConn DC-Stimulator Plus device (neuroConn Technology). Stimulating electrodes were dampened in saline and placed under the EEG cap at positions CP5 and FC1 (10–20 system; Figure 1D). Alpha tACS was applied intermittently at participants’ individual alpha frequency at 1 mA, for a period of 6 s on/off (every other trial; Figure 1E, top). This amounted to 7.5 min of total stimulation (150 trials, “during” tACS block). Sham tACS was applied at 1 Hz, ramped up to 1 mA for one cycle and then turned off. It was also applied intermittently (at the beginning of every other trial; Figure 1E, bottom) in order to replicate any potential physical sensation caused at the onset of electrical stimulation.

### EEG Data Acquisition

Thirty channels (two channels were removed for stimulating electrodes) of EEG data were sampled at 30 kHz using the open-source Open Ephys + EEG system (Black et al., 2017; Siegle et al., 2017). This system allowed us to switch the amplifier on/off rapidly during tACS in order to prevent signal saturation. The electrode cap configuration was taken from a standard 10–20 system layout (Figure 1D), using active electrodes for noise cancellation from a Brain Vision actiCap. Raw EEG data were down-sampled to 250 Hz, and filtered from 1 to 50 Hz (high- and low-pass Butterworth filters). Signals were subsequently cleaned using standard methods implemented in EEGLAB (Delorme and Makeig, 2004); noisy channels were removed, signals were re-referenced to the common average, epochs were extracted from -1,000 to 1,000 ms (relative to the tactile stimulus at *t* = 0), outliers were identified and removed via visual inspection, and independent component analysis was performed with the *fastica* algorithm (Bingham and Hyvärinen, 2000) to remove biological artifacts, such as eye-blinks.

All EEG data presented here represent signals from the C3 electrode, located over somatosensory cortex contralateral to the tactile stimulus (Figure 1D). All EEG analysis was performed on trials in which threshold-level tactile stimuli were delivered. EEG data from supra-threshold and null trials were excluded from analysis due to a lower trial count (10% and 20% of trials, respectively) compared to threshold-level trials (70% of trials). To assess time and frequency domain EEG correlates of somatosensory perception, all trials incorporating threshold-level tactile stimuli were separated into perceived (“hit”) and non-perceived (“miss”) trials. To compare effects of tACS with sham electrical stimulation, data were separated into pre- and post-electrical stimulation time blocks (i.e., ‘pre-tACS’ and ‘post-tACS’).

### EEG Spectral Analysis

Spectral analysis was carried out using methods consistent with our previous work (Jones et al., 2009; Sherman et al., 2016; Shin et al., 2017). Data were analyzed during the pre-stimulus (tactile stimulus) time period; each trial was defined within a window from -1,000 to 0 ms relative to tactile stimulus onset. Spectrograms were calculated by convolving the clean EEG signal with a complex Morlet wavelet of the form:

$$w(t,\;f_{0})=A\;\exp\left(-\frac{t^{2}}{2\sigma_{t}^{2}}\right)\exp(2i\pi f_{0}t)$$

for each frequency of interest *f*_0_ at time *t* between 1 and 50 Hz, where σ = *m*/2π*f*_0_, *i* is the imaginary unit and $A=1/\sigma_{t}\sqrt{2\pi }$ is the normalization factor.

The number of Morlet wavelet cycles (*m*) was set as a constant of 7, consistent with prior work in the lab (Jones et al., 2009, 2010; Ziegler et al., 2010; Wan et al., 2011; Sacchet et al., 2015; Shin et al., 2017). Time–frequency representations of power (TFRs) were calculated as the squared magnitude of the complex wavelet-convolved data. TFR values were then normalized by the median power value for each frequency. This median was calculated from all power values, at each frequency, in the -1,000 to 0 ms pre-stimulus TFR concatenated across trials.

Normalized TFR values were calculated in factors of median (FOM) for each frequency, separately for each subject/session. To calculate mean pre-stimulus power for each trial, normalized TFR values were averaged across time (-1,000 to 0 ms) and frequencies within the corresponding band. For the alpha frequency band, individual peak alpha frequencies were identified as the maximum value within a band from 7 to 14 Hz, and subsequently averaged over a frequency band encompassing ±2 Hz from this maximum value. Maximum values were identified for the pre- and post-stimulation time blocks separately.

### EEG Evoked Response

Baseline correction was applied separately for each trial. Mean voltage values within a time window from -150 to 0 ms were subtracted from values at each time-point within each trial. Mean evoked responses were then calculated by averaging voltage values (from 0 to 1,000 ms, relative to stimulus onset) across trials for each subject, and across subjects.

### Computational Neural Modeling Using Human Neocortical Neurosolver (HNN)

Our group has developed a unique biophysically principled computational neural model of the neocortical circuitry that links human EEG or MEG signals to underlying cellular and circuit-level mechanisms, based on their biophysical origin (Jones et al., 2007, 2009; Ziegler et al., 2010; Lee and Jones, 2013; Sherman et al., 2016; Neymotin et al., 2018). The model represents a laminated cortical column with synaptically coupled inhibitory interneurons and excitatory neurons across layers (i.e., basket cells and pyramidal neurons), and includes exogenous excitatory synaptic drive to distinct layers. It also simulates the primary electrical current activity (i.e., current dipoles) that generates EEG/MEG sensor data from post-synaptic intracellular currents in large and spatially aligned dendrites of cortical pyramidal neurons (Hämäläinen et al., 1993; Okada et al., 1997; Murakami and Okada, 2006; see Figure 9A). The model has been applied previously to study the origin of somatosensory oscillations and tactile evoked responses (Jones et al., 2007, 2009; Ziegler et al., 2010; Lee and Jones, 2013; Sherman et al., 2016).

Our group has recently developed this model into a user-friendly software tool named Human Neocortical Neurosolver (HNN). HNN allows for the development and testing of specific hypotheses regarding the neural origins of EEG/MEG signals using a graphical user interface (GUI; Neymotin et al., 2018). A detailed description, tutorials, and open-source freely available distribution of this tool can be found at http://hnn.brown.edu.

For the purposes of the analysis presented here, we utilized the model parameters associated with the ‘ERP tutorial’ on the HNN website^1^ (see Supplementary Materials). This tutorial provides a parameter set that simulates a SI-localized MEG evoked response potential (ERP) elicited by threshold-level taps to the finger, analogous to the tactile stimulation paradigm used in the current study. The ERP is simulated using a layer-specific sequence of exogenous excitatory synaptic drive to the local SI circuitry (as described in Jones et al., 2007, 2009). This sequence of drive reproduces early tactile evoked response peaks up to 165 ms post-stimulus (see Supplementary Figure S1). We specifically modified the model parameters representing synaptic gain, which can be accessed through the HNN GUI. This allows for adjustment of excitatory and inhibitory synaptic conductance within the model network by multiplying the targeted synaptic conductance weights (inhibitory:GABAA/GABAB or excitatory: AMPA/NMDA, respectively) by a specified amount. To test the specific hypothesis that tACS can affect synaptic plasticity, we increased total synaptic gain (i.e., both inhibitory and excitatory synaptic conductance weights), as well as inhibitory and excitatory synaptic gain parameters alone, by a factor of 2. Simulated evoked responses under various gain changes were compared with recorded EEG data to interpret potential effects of tACS on the EEG evoked response.

All simulations in this study can be reproduced by downloading the HNN software, running the ERP tutorial, and changing the synaptic gain parameters, as described. Parameter files used in the current analysis are included in the Supplementary Materials).

## Results

### EEG Correlates of Threshold-Level Tactile Detection

**FIGURE 2:**
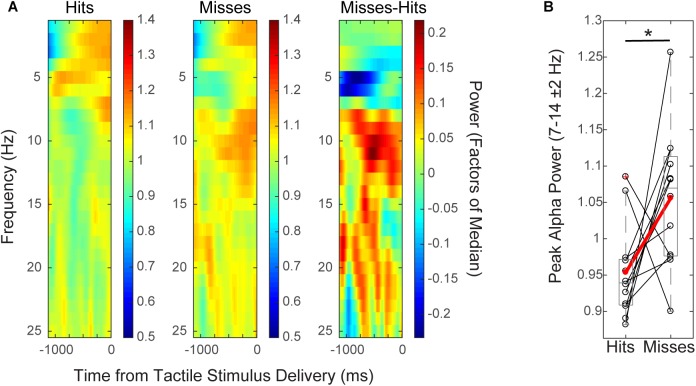
Mean pre-stimulus EEG alpha power is higher on non-perceived trials. **(A)** Spectrogram shows time–frequency representation (TFR) values averaged across participants over the pre-stimulus time period (–1 to 0 s), for hit and miss trials (left, middle), and for the difference between conditions (miss-hit trials, right). **(B)** Mean pre-stimulus alpha power (FOM; individual peak power between 7–14 ± 2 Hz) for individual subjects (black), and averaged across subjects (red) shows that alpha power prior to the tactile stimulus is higher on non-perceived (“miss”) than perceived (“hit”) trials (Wilcoxon signed rank test, ^∗^*p* < 0.05). Only data from the pre-tACS time block is included in this analysis.

**FIGURE 3:**
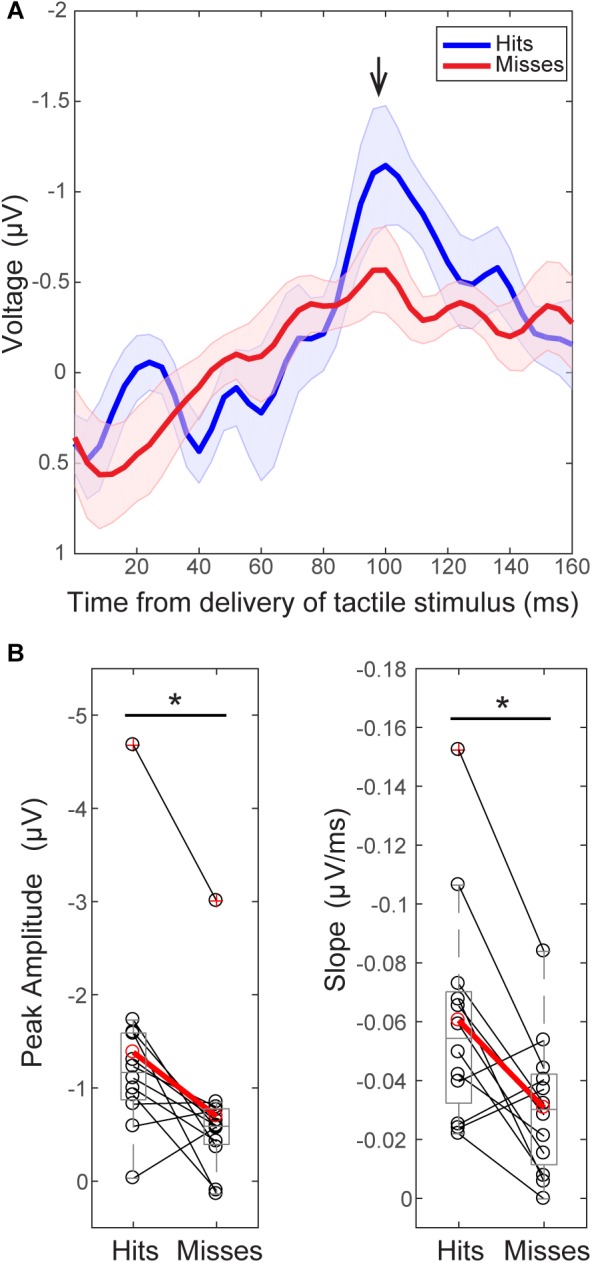
The tactile stimulus-evoked response differs on perceived and non-perceived trials. **(A)** Grand average EEG (C3 electrode) evoked responses (±SEM) following delivery of the tactile stimulus (*t* = 0) for all trials during the pre-tACS time block. Black arrow indicates the prominent peak ∼100 ms post-stimulus (minimum value between 92–108 ms, 98.5 ± 0.15 ms). **(B)** Peak values ∼100 ms were significantly higher on perceived (“hit”, above in blue) than non-perceived (“miss”, above in red) trials across subjects [left, Wilcoxon signed ranks test (WSR), ^∗^*p* < 0.05]. The mean slope from ∼70 ms (peak value between 60 and 90 ms, 74 ± 1.6 ms) to ∼100 ms was also significantly larger on perceived (blue, “hit”) than non-perceived (red, “miss”) trials (right, WSR, ^∗^*p* < 0.05). Only data from pre-tACS block is included in this analysis.

In previous MEG studies, we showed that averaged pre-stimulus (-1,000 to 0 ms) alpha power in SI (source-localized) was higher on trials when threshold-level tactile stimuli were not perceived, compared to trials in which they were perceived (Jones et al., 2010). Correlates of perception were also found in the early period (0–175 ms) of the post-stimulus tactile evoked response, such that ERP peaks were larger and emerged sooner on perceived trials (Jones et al., 2007). Here, we employed an analogous tactile detection task. Prior to employing tACS, we tested whether EEG-measured, sensor-level (C3) signals reflect these same perceptual differences (i.e., pre-tACS time blocks only; Figures 2, 3).

#### Mean Pre-stimulus EEG Alpha Power Is Higher on Non-perceived, Than Perceived Trials

Group level time–frequency spectrograms (Figure 2A) and peak alpha power values from individual subjects (Figure 2B) from EEG sensor (C3) data demonstrated that normalized mean pre-stimulus alpha power (FOM; -1 to 0 s from tactile stimulus; peak frequency between 7–14 ± 2 Hz) across subjects was significantly higher on non-perceived (“miss”) trials than on perceived (“hit”) trials (Figure 2; mean -1,000 to 0 ms prior to stimulus onset; *n* = 12, Wilcoxon signed rank test (WSR), *p* = 0.041, Z = -2.040; Hits: Mean = 0.95, SEM = 0.06 μV^2^; Misses: Mean = 1.06, SEM = 0.03 μV^2^). There was also a trend toward significance in the beta band (peak frequency 15–29 ± 2 Hz; WSR, *p* = 0.099, *Z* = -1.647; Hits: Mean = 0.99, SEM = 0.01 μV^2^; Misses: Mean = 1.03, SEM = 0.02 μV^2^). Normalized mean pre-stimulus power averaged over theta and gamma frequency bands did not differ significantly between perceived and non-perceived trials (data not shown; Theta: peak frequency between 4–7 ± 2 Hz; *n* = 12; WSR, *p* = 0.433, *Z* = -0.784; Hits: Mean = 1.01, SEM = 0.03 μV^2^; Misses: Mean = 0.95, SEM = 0.04 μV^2^; Gamma: peak frequency between 30–50 ± 2 Hz; *n* = 12; WSR, *p* = 0.814, *Z* = -0.235; Hits: Mean = 1.04, SEM = 0.03 μV^2^; M = 1.02, SEM = 0.02 μV^2^). Data were collapsed across sham and alpha tACS group participants during the pre-electrical stimulation time block only.

#### The Tactile Stimulus-Evoked EEG Response Differs on Perceived and Non-perceived Trials

In further agreement with our prior MEG studies (source-localized SI; Jones et al., 2007), grand average sensor-level EEG evoked responses following delivery of the perceptual threshold-level tactile stimulus showed significant differences between perceived and non-perceived trials (Figure 3; pre- alpha/sham tACS trials only).

For consistency in interpreting the current results in relation to our prior reports (Jones et al., 2007, 2010), *y*-axes of the EEG evoked response values in Figures 3A, 8A, and 9E are flipped so that the apparent sign of the ∼100 ms peak is positive (black arrow, Figure 3), consistent with source-localized MEG data. This adjustment of the sensor and source peaks allowed for a comparable interpretation of the circuit-level dynamics underlying the tactile evoked response, as identified in our prior studies (Jones et al., 2007, 2009). It is important to note that the signal to noise ratio in EEG sensor data is smaller than in source-localized MEG signals. Therefore, early components of the threshold-level evoked response (<100 ms post-stimulus) were difficult to distinguish in the current EEG dataset, particularly in non-perceived trials. However, the timing of the ∼100 ms evoked response peak, and perceptual differences in evoked response features, were remarkably consistent across recording modalities, as described below.

As in our prior report (Jones et al., 2007), two main differences were observed between averaged ERPs from detected (Figure 3; “hits,” blue) and non-detected (“misses,” red) trials. First, peak values near ∼100 ms (minimum value between 92–108 ms, Mean = 98.5 ms, SEM = 1.15 ms) post-stimulus were significantly larger in magnitude on perceived than non-perceived trials (Figure 3B, left; *n* = 12; WSR, *p* = 0.015, *Z* = -2.432; Hits: Mean = -1.38, SEM = -0.33 μV; Misses: Mean = 0.70, SEM = 0.23 μV). Second, the mean slope from ∼70 ms (maximum peak between 60–88 ms) to ∼100 ms (minimum peak between 92–108 ms) was significantly higher on perceived than non-perceived trials across subjects (Figure 3B, right; *n* = 12; WSR, *p* = 0.010, *Z* = -2.589; Hits: Mean = -0.06, SEM = 0.01 ms/μV; Misses: Mean = -0.03, SEM = 0.01 ms/μV). Peak values near ∼70 ms (maximum 60–88 ms, Mean = 74.4 ms, SEM = 1.64 ms) post-stimulus were not significantly different on perceived and non-perceived trials (data not shown; *n* = 12; WSR, *p* = 0.754, *Z* = -0.314; Hits: Mean = 0.12, SEM = 0.33 μV; Misses: Mean = 0.01, SEM = 0.19 μV).

### Effects of tACS

Having demonstrated consistency in EEG and MEG results (see Jones et al., 2007) relating tactile perception to pre-stimulus alpha power and post-stimulus evoked responses, we sought to characterize potential effects of alpha frequency tACS on perception, and the above established EEG correlates of perception (i.e., differences in pre-stimulus alpha power and ERP waveforms).

#### Tactile Detection Performance Decreases Over Time

**FIGURE 4:**
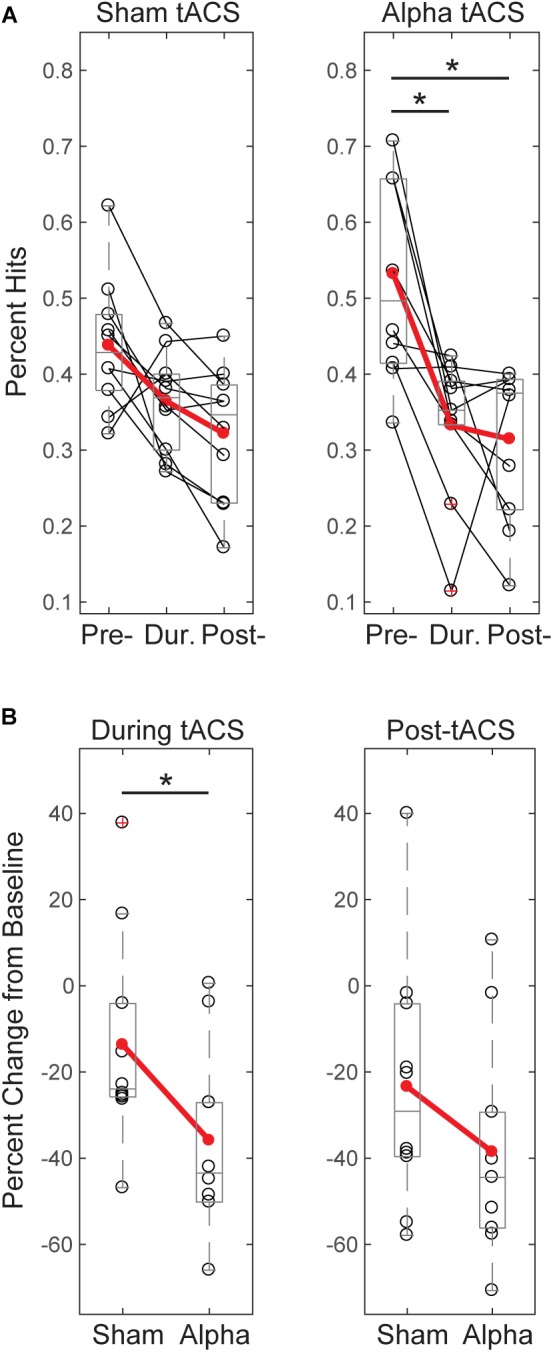
Tactile detection performance decreases over time and differs between groups during tACS. **(A)** Individual participants’ mean performance (percent hits; black circles) on the tactile detection task before, during and after sham or alpha stimulation. Red circles denote group averages. Performance decreased over time for both sham and alpha tACS groups, with significant differences in performance between pre- and during, and pre- and post-alpha tACS time blocks (Friedman test, *p* < 0.05; *post-hoc* Wilcoxon signed ranks tests with Bonferroni correction, ^∗^*p* < 0.01). **(B)** Individual participants’ (black circles) and group averaged (red circles) percent change in performance from baseline shows that participants who received alpha tACS did significantly worse on the task compared to sham during the tACS time block (*n* = 10/group, Mann–Whitney *U* test, ^∗^*p* < 0.05), but not afterward.

Figure 4A shows individual subjects’ behavioral performance in the tactile detection task before, during, and after either sham or individualized alpha tACS. Percent hits were calculated as total hits/total trials for threshold-level trials only. We tested for a main effect of time on tactile detection performance and found that both groups’ performance decreased across time. After correcting for multiple comparisons, the alpha tACS group exhibited significant reductions in behavior from the “pre-” to “during” tACS time blocks, and from “pre-” to “post-tACS” time blocks. The sham group trended toward significance from “pre- to post-,” and “during to post-” tACS time blocks, but did not survive correction for multiple comparisons (Friedman test; Sham: χ^2^= 8.600(2), *p* = 0.014; Pre mean = 0.44, SEM = 0.03, During mean = 0.36, SEM = 0.02, Post mean = 0.32, SEM = 0.03; Alpha tACS: χ^2^= 11.556(2), *p* = 0.003; Pre mean = 0.53, SEM = 0.04, During mean = 0.36, SEM = 0.02, Post mean = 0.31, SEM = 0.04; *Post hoc* WSR with Bonferroni corrected significance level set to *p* < 0.017; Alpha tACS: Pre/During p = 0.007, *Z* = -2.703; Pre/Post *p* = 0.009, *Z* = -2.601; During/Post *p* = 0.540, *Z* = -0.612; Sham: Pre/During: *p* = 0.066, *Z* = -1.836; Pre/Post: *p* = 0.022, *Z* = -2.295; During/Post *p* = 0.028, *Z* = -2.191).

#### Tactile Detection Performance Differs Between Groups During Alpha tACS

By chance, initial performance appeared higher, and variance in behavior appeared larger, in the pre-alpha tACS group than sham (Figure 4A). Therefore, when comparing between groups, we first assessed whether the alpha tACS group had higher baseline performance by comparing pre-tACS task performance. There was no significant difference in mean percent hits between pre-sham vs. pre-alpha conditions [*n* = 20; Mann–Whitney *U* test (MWU), *p* = 0.053, *Z* = -1.966; Sham: Mean = 43.7, SEM = 2.8 μV^2^; Alpha: Mean = 52.3, SEM = 11 μV^2^], and the distributions were not significantly different (Kolmogorov–Smirnov test *p* > 0.05). Accordingly, performance during and post-tACS was normalized by pre-tACS performance (percent change from baseline). During tACS, the reduction in normalized task performance was significantly greater in the alpha tACS group compared to the sham group (Figure 4B, left panel), but the effect did not last into the post-tACS time block (Figure 4B, right panel; During tACS: MWU, *n* = 6/group; *p* = 0.023, *Z* = -2.269, Sham: Mean = -13.72, SEM = 7.76; Alpha: Mean = -35.91, SEM = 6.76; Post-tACS: MWU, *n* = 6/group; *p* = 0.190, *Z* = -1.324; Sham: Mean = -23.45, SEM = 9.30; Alpha: Mean = -38.56, SEM = 8.03).

#### Threshold-Level Stimulus Intensity Does Not Change Over Time or Differ Between Groups

**FIGURE 5:**
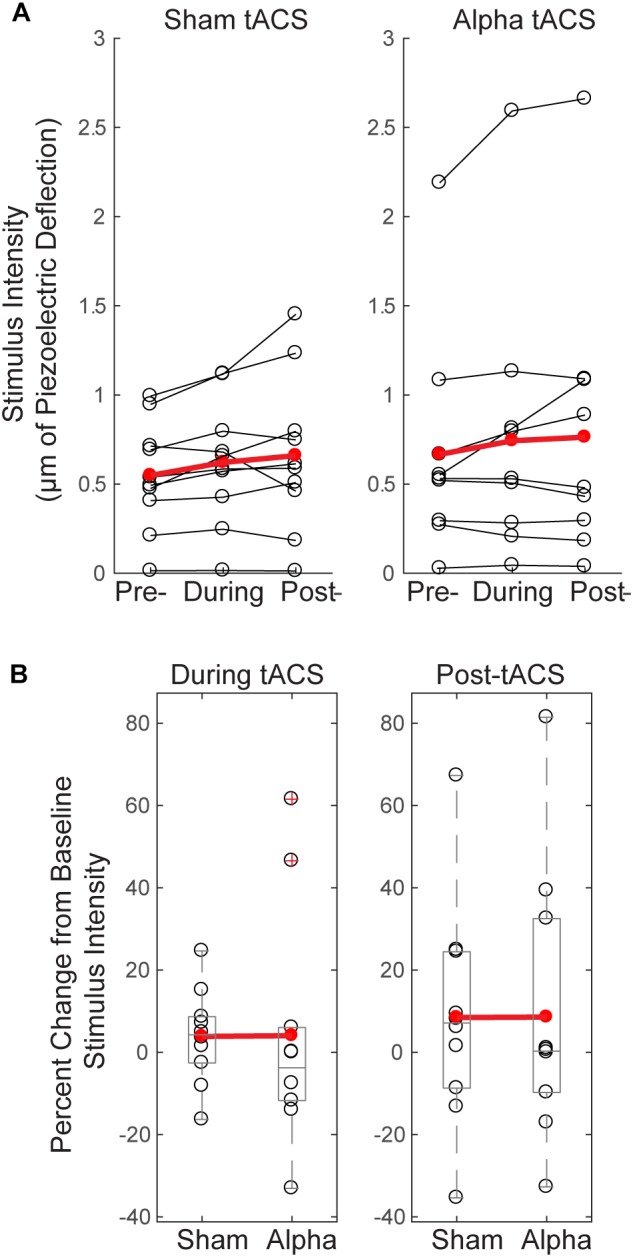
Threshold-level stimulus intensity does not change over time or differ between groups. **(A)** Tactile stimulus intensity remained relatively constant over the course of the experiment (pre-, during and post-tACS) for both groups (Friedman test, *p* > 0.05), despite dynamic modulation of stimulus intensity (see “Materials and Methods”). **(B)** Baseline normalized tactile stimulus intensities did not differ between sham and alpha tACS groups during or after electrical stimulation (Mann–Whitney *U* test, *p* > 0.05).

Since tactile stimulus intensity was modulated dynamically throughout the detection task (see “Materials and Methods”), and there were effects of tACS on behavior (Figure 4), we tested whether these effects where accompanied by changes in tactile stimulus intensity over time, or between groups. Figure 5A demonstrates that there were no significant changes in stimulus intensity over time (measured in μm of piezoelectric deflection; pre-, during and post-tACS) for either sham or alpha tACS groups (Friedman test; Sham: χ^2^= 2.513(2), *p* = 0.285; Pre mean = 246, SEM = 43; During mean = 256, SEM = 44; Post mean = 266, SEM = 46; Alpha: χ^2^= 0.22(2), *p* = 0.895; Pre mean = 242, SEM = 43; During mean = 243, SEM = 47; Post mean = 260, SEM = 52).

Figure 5B shows mean tactile stimulus intensities during and after electrical stimulation, normalized by pre-tACS intensity (percent change from baseline). There was no significant difference in normalized mean tactile stimulus intensity between sham and alpha tACS groups during or after electrical stimulation (During tACS: MWU, *n* = 20; *p* = 0.393, *Z* = -0.907; Alpha: Mean = 4.05, SEM = 9.0; Sham: Mean = 3.87, SEM = 3.64; Post-tACS: MWU, *n* = 20; *p* = 0.631, *Z* = -0.529; Alpha: Mean = 8.57, SEM = 10.60; Sham: Mean = 8.46, SEM = 8.64).

#### There Is no Measurable Difference in Alpha Power Before and After tACS

**FIGURE 6:**
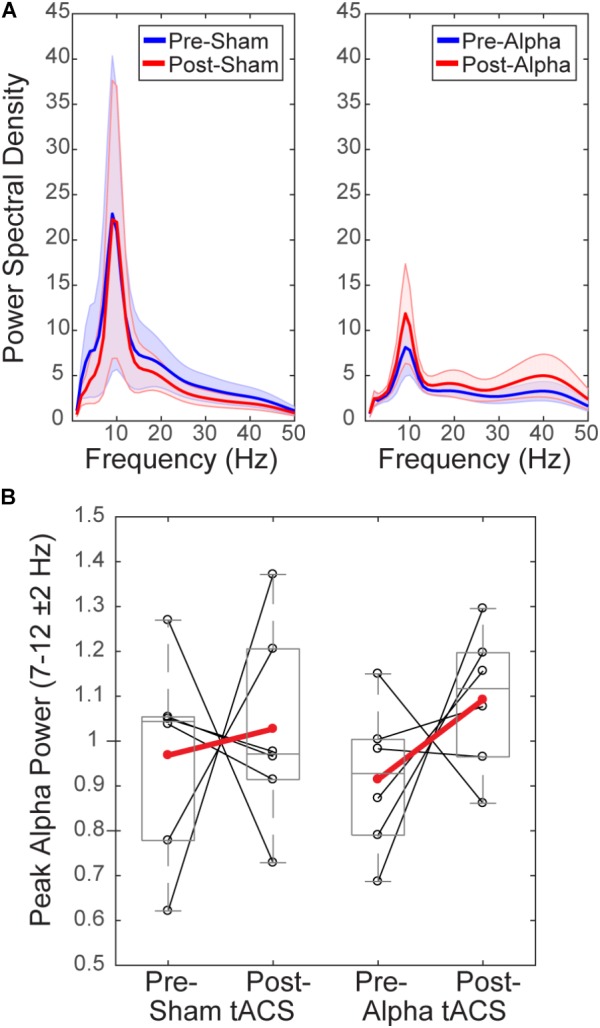
Mean pre-stimulus alpha power is not different before and after tACS. **(A)** Power spectral density (PSD) plots show mean time–frequency representation (TFR) values (±SEM) over the 1 s time period prior to delivery of the tactile stimulus (–1–0 s), averaged across participants, for frequencies within the alpha range (individual peak frequencies between 7–14 ± 2 Hz). **(B)** Normalized pre-stimulus alpha power averaged across the pre-stimulus time period is shown for individual subjects (black circles), and averaged within groups (red circles), before and after sham and alpha tACS. Mean pre-stimulus alpha power did not differ significantly within groups (pre- vs. post-) for alpha or sham tACS groups (*n* = 6/group, Wilcoxon signed ranks test, *p* > 0.05). There were also no significant differences in mean pre-stimulus alpha power between groups (sham vs. alpha) before or after tACS (Mann–Whitney *U* test, *p* > 0.05).

To test whether tACS induced measurable lasting differences in alpha rhythms, we compared normalized mean alpha power (FOM; individual peak power between 7–14 ± 2 Hz) within a 1 s time window prior to the tactile stimulus (‘pre-stimulus’; -1 to 0 s), averaged over time within each of the pre- vs. post- sham/alpha tACS time blocks (Figure 6). Group level power spectral density plots (Figure 6A) and individual subjects’ results (Figure 6B) show that mean pre-stimulus alpha power was not significantly different within groups between pre- and post-sham, or pre- and post-alpha tACS conditions (WSR, *n* = 6/group; Sham: *p* = 0.917, *Z* = -0.105; Pre: Mean = 0.97, SEM = 0.09 μV^2^; Post: Mean = 1.03, SEM = 0.09 μV^2^; Alpha: *p* = 0.249, *Z* = -1.153; Pre: Mean = 0.91, SEM = 0.07 μV^2^; Post: Mean = 1.09, SEM = 0.06 μV^2^). There was also no significant difference in pre-stimulus alpha power between groups for pre-sham vs. pre-alpha tACS, or post-sham vs. post-alpha tACS conditions (*n* = 12; MWU Pre: *p* = 0.589, *Z* = -0.641; Sham: Mean = 0.97, SEM = 0.09 μV^2^; Alpha: Mean = 0.91, SEM = 0.07 μV^2^; Post: *p* = 0.818, *Z* = -0.320; Sham: Mean = 1.03, SEM = 0.09 μV^2^; Alpha: Mean = 1.09, SEM = 0.06 μV^2^). We conclude that tACS did not induce clear persistent changes in alpha power. However, since we were unable to analyze EEG activity during the tACS time block, we could not directly examine whether EEG alpha oscillations were entrained during alpha stimulation (see “Discussion”).

#### Baseline Alpha Power Influences the Change in Alpha Power Over Time

**FIGURE 7:**
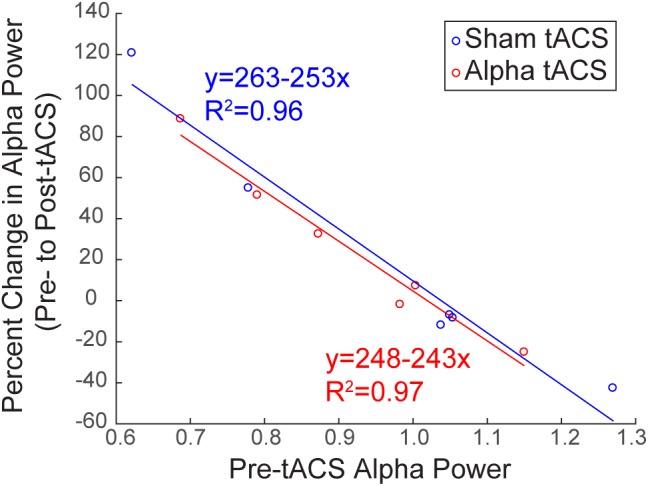
Baseline alpha power influences the change in alpha power over time. There was a significant inverse linear relationship between pre- and post-tACS alpha power. Lower pre-tACS alpha power predicted an increase in power post-tACS, whereas higher pre-tACS alpha power predicted a decrease in power post-tACS, for both sham (blue; linear regression, ^∗^*p* = 0.001, *R*^2^= 0.96) and alpha (red, linear regression, ^∗^*p* < 0.001; *R*^2^= 0.97) tACS groups.

Several studies have demonstrated that modulation of rhythmic activity is brain state-dependent (e.g., Alagapan et al., 2016). In particular, low endogenous alpha activity may be a necessary precondition for modulation of alpha power using NIBS (Lefebvre et al., 2017). Since we observed both increases and decreases in alpha power following tACS (Figure 6B), we tested whether there was a relationship between pre-tACS alpha power and the change in alpha power from pre- to post-tACS time blocks (Figure 7). Linear regression established that pre-tACS alpha power (FOM; individual peak power between 7–14 ± 2 Hz) was inversely correlated with the change in alpha power from pre- to post-tACS time blocks, for both sham and alpha tACS groups [Figure 7; Sham: *F*(1,4) = 86.65, *p* = 0.001, *R*^2^= 0.956; Alpha: *F*(1,4) = 124.16, *p* < 0.001, *R*^2^= 0.969]. In both groups, low pre-tACS alpha power resulted in an increase in alpha power post-tACS, whereas high pre-tACS alpha power resulted in a decrease in alpha power post-tACS. Specifically, if normalized pre-tACS alpha power was <∼1, power increased over time, whereas if normalized pre-tACS alpha power was >∼1, power decreased over time (Figure 7; see also Figure 6B).

#### Emergence of a 70 ms Evoked Response Peak Following Alpha-tACS

**FIGURE 8:**
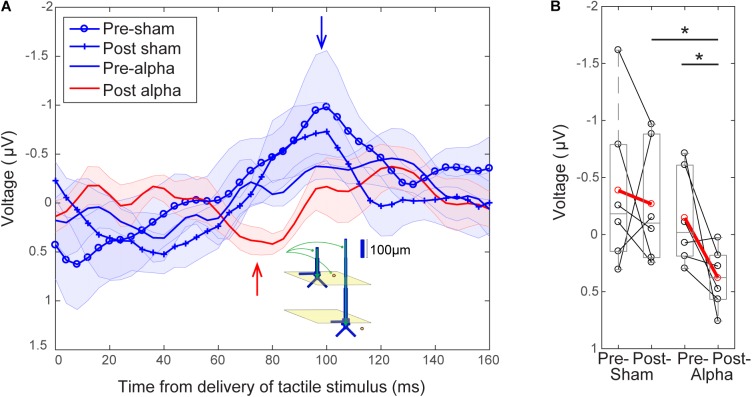
Emergence of a ∼70 ms evoked response peak following tACS. **(A)** Grand average EEG evoked responses averaged over tACS conditions (±SEM). Waveforms following delivery of the tactile stimulus (at *t* = 0) all have a relatively uniform shape in the absence of alpha tACS. Pre-sham (blue circles), post-sham (blue crosses) and pre-alpha (blue line) tACS evoked response waveforms possess a characteristic peak ∼100 ms post-stimulus (blue arrow). The post-alpha tACS waveform (red line) has a lower amplitude peak at ∼100 ms, and is marked by the emergence of a downward peak ∼70–80 ms post-stimulus (red arrow). Inset illustrates a potential circuit interpretation of the ∼70–80 ms peak, based on prior computational neural modeling (see “Discussion”). **(B)** The mean magnitude of the signal near the ∼70–80 ms peak (averaged between 68–84 s) was significantly larger in the post-alpha tACS condition than in both the pre-alpha (Wilcoxon signed ranks test, ^∗^*p* < 0.05) and post-sham (Mann–Whitney *U* test, ^∗^*p* < 0.05) tACS conditions.

Finally, we investigated whether alpha tACS visibly changed the grand average evoked response at electrode C3 (Figure 8). In the absence of alpha tACS, tactile evoked response waveforms had a relatively uniform shape, marked by a prominent peak at ∼100 ms post-stimulus (blue traces and arrow, Figure 8A). The post-tACS evoked response waveform was marked by the apparent emergence of another prominent peak ∼70–80 ms post-stimulus (red trace and arrow, Figure 8A), which was not clearly discernible in the pre-sham, post-sham or pre-alpha tACS subject averages. In prior studies using MEG, a similar peak near 70 ms (named the M70) was reliably observed in signals source localized to SI, following sub- and supra-threshold level taps to the finger (Jones et al., 2007, 2009, see also Supplementary Figure S1). Tactile evoked response peaks between ∼60 and 80 ms have also been consistently observed in other MEG and EEG studies (Forss et al., 1994; Hoechstetter et al., 2001; Druschky et al., 2003; Zhang and Ding, 2010), as well as in invasive LFP recordings in monkeys (Kulics, 1982; Kulics and Cauller, 1986; Cauller and Kulics, 1991; Lipton, 2006). Further, computational neural modeling has been applied to interpret the circuit origin of the M70 peak, as well as differences in its magnitude with perception (Jones et al., 2007, see the sections “Computational Neural Modeling Suggests Enhanced Synaptic Gain Can Account for Post-tACS Evoked Response Differences” and “Discussion”). Due to its robust emergence in tactile processing, we quantified the difference in magnitude of peaks around ∼70–80 ms across stimulation conditions in our EEG data in order to see if the peak was specifically affected by tACS. To do so, mean signal magnitudes within a time window encompassing the emergent peak (68–84 ms post-stimulus) were compared across stimulation conditions.

Figure 8B shows that the mean magnitude of the signal encompassing the ∼70–80 ms peak was significantly larger in the post-alpha tACS condition than in both pre-alpha and post-sham tACS conditions, but not significantly different between pre- and post-sham tACS, or between pre- alpha and pre-sham tACS conditions (Pre- vs. Post-Alpha: mean 68–84 ms, *n* = 6; WSR, *p* = 0.046, *Z* = -1.992; Pre: Mean = -0.15, SEM = 0.17 μV; Post: Mean = 0.38, SEM = 0.11 μV; Post-Alpha vs. Sham: mean 68–84 ms, *n* = 12; MWU, *p* = 0.026, *Z* = -2.242; Alpha: Mean = 0.38, SEM = 0.11 μV; Sham: Mean = -0.2675, SEM = 0.2167 μV; Pre- vs. Post-sham: mean 68–84 ms, *n* = 6; WSR, *p* = 0.600, *Z* = -0.524; Pre: Mean = -0.39, SEM = 0.29 μV; Post: Mean = -0.27, SEM = 0.22 μV; Pre- Alpha vs. Sham: *n* = 12; MWU, *p* = 0.699, *Z* = -0.480; Alpha: Mean = -0.15, SEM = 0.17 μV; Sham: Mean = -0.39, SEM = 0.29 μV).

The magnitude of the ∼100 ms peak was not significantly different between pre- and post-alpha tACS, or between pre- and post-sham tACS conditions (data not shown; Alpha: *n* = 6; WSR, *p* = 0.753, *Z* = -0.314; Pre: Mean = -0.50, SEM = 0.12 μV; Post: Mean = 0.32, SEM = 0.26 μV; Sham: *n* = 6; WSR, *p* = 0.600, *Z* = -0.524; Pre: Mean = -1.08, SEM = 0.55 μV; Post: Mean = -0.82, SEM = 0.29 μV).

### Computational Neural Modeling Suggests Enhanced Synaptic Gain Can Account for Post-tACS Evoked Response Differences

**FIGURE 9:**
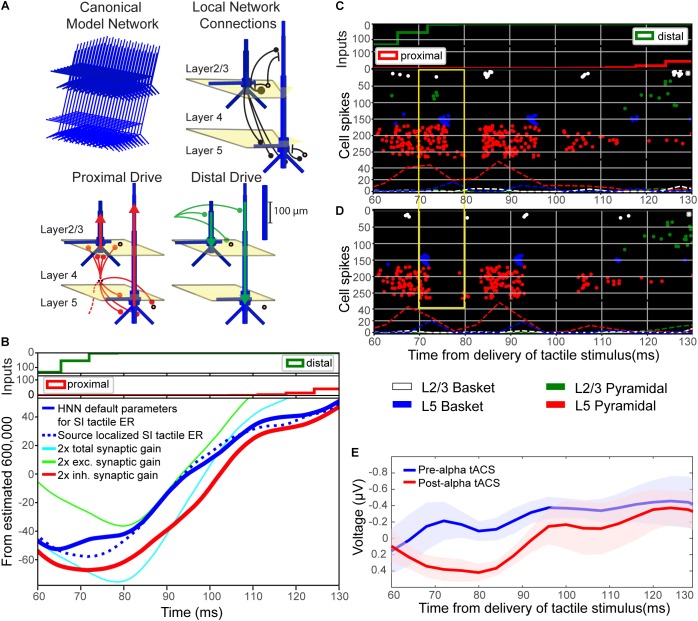
Computational neural modeling suggests enhanced synaptic gain can account for the post-tACS evoked response. **(A)** Schematic illustration of the primary somatosensory cortex (SI) model network underlying the Human Neocortical Neurosolver (HNN) software. The network is represented by a canonical model of a layered neocortical column (top left), with synaptically coupled inhibitory (yellow circles, basket cells) and excitatory neurons (blue, multi-compartment pyramidal neurons) across layers (top right). It also includes two distinct pathways of exogenous excitatory synaptic network inputs that effectively drive proximal (red) and distal (green) dendrites of the cortical pyramidal neurons (bottom panels). HNN simulates the primary electrical currents underlying EEG/MEG signals (red and green arrows) from net post-synaptic intracellular currents within the large and spatially aligned cortical pyramidal neuron dendrites (see “Materials and Methods” for further details). **(B)** Simulation of the tactile evoked response from 60 to 130 ms (see Supplementary Figure S1 for additional description of the tactile evoked response simulation). A default model simulation of the SI threshold-level tactile evoked response (solid blue line), provided in HNN, was tuned to MEG data from a prior study (dotted blue line; Jones et al., 2007). Increasing local synaptic gain (i.e., maximal conductance of excitatory and inhibitory synapses) by a factor of 2 in the model simulation (cyan line) reproduced the enhanced ∼70–80 ms peak observed post-tACS. Increasing synaptic gain separately for excitatory or inhibitory populations, respectively, demonstrated that the simulated ∼70–80 ms peak was driven mainly by enhanced inhibitory synaptic gain (red line; compare to **E**), as opposed to enhanced excitatory synaptic gain (green line). **(C,D)** Spiking activity of each cell in the network **(C)** before and **(D)** after modifying synaptic gain demonstrates that increasing total synaptic gain resulted in decreased firing of layer 5 pyramidal neurons (red circles, yellow box) around the same time as the enhanced ∼70–80 ms peak. **(E)** EEG tactile evoked responses from 60 to 130 ms during pre- and post-tACS time blocks. The pre-alpha tACS EEG evoked response (blue line) closely resembles the simulation of a threshold-level tactile evoked response (compare to solid blue line, **B**), while the post-alpha tACS EEG evoked response most closely resembles the model simulation with enhanced inhibitory synaptic gain (compare to red line, **B**).

Motivated by prior studies investigating the circuit-level impact of NIBS directly on synaptic dynamics (e.g., Kronberg et al., 2017; Rahman et al., 2017), we applied a computational neural modeling tool designed by our group to interpret the circuit origin of MEG/EEG signals (Human Neocortical Neurosolver: HNN, see “Materials and Methods”) in order to investigate the source of the observed emergence of the ∼70–80 ms evoked response peak in the post-alpha tACS time period (Figure 8). We used our model (Figure 9A; Jones et al., 2007, 2009) to test the specific hypothesis that alpha tACS may affect synaptic plasticity, as suggested by others (Zaehle et al., 2010; Polanía et al., 2012; Vossen et al., 2015; Lafon et al., 2017). We tested this hypothesis by simulating the tactile evoked response in the pre-tACS condition, and then changing the local synaptic gain (i.e., local excitatory and inhibitory synaptic conductance) to see if these changes could account for the emergence of the ∼70–80 ms post-tACS peak. Figure 9B shows model simulated evoked responses near the ∼70–80 ms peak (60–130 ms post-stimulus), and Figure 9E shows corresponding evoked responses in the pre- and post-alpha tACS EEG data.

In the model, the downward deflection in the waveform near ∼70–80 ms (M70 in Jones et al., 2007) is created by excitatory synaptic drive to the distal dendrites, while the subsequent rise between 100 and 130 ms is created by pyramidal cell spiking (Figure 9C) and re-emergent excitatory proximal drive near 130 ms. Histograms of spike patterns providing the exogenous proximal and distal drive are shown at the top of Figure 9B (see also Supplementary Figure S1). This pattern of input is consistent with prior reports and laminar recordings in animals, and can thus be interpreted as a “feedback” input from a higher order cortical region (presumably SII) at 70 ms, followed by a re-emergent thalamic input at ∼130 ms (Cauller and Kulics, 1991; see also prior studies Jones et al., 2007, 2009 and Supplementary Figure S1).

Visual inspection revealed that the pre-tACS evoked response (blue trace, Figure 9E) could be reproduced with the HNN default evoked response parameter set (blue solid trace, Figure 9B; see Supplementary Materials) that simulates the SI tactile evoked response based on prior MEG studies (blue dotted trace, Figure 9B; MEG data is also provided with the HNN software). Doubling both the excitatory and inhibitory synaptic gain in the model simulation reproduced the enhanced ∼70–80 ms peak observed in our post-tACS EEG data (cyan curve, Figure 9B). This result is consistent with the hypothesis that local synaptic facilitation could account for the observed EEG correlates of alpha tACS. More specifically, tACS may amplify the impact of distal “feedback” input (∼70 ms post-stimulus) to the SI circuitry.

Simulating changes in gain separately for excitatory (green curve, Figure 9B) or inhibitory (red curve, Figure 9B) synapses, respectively, demonstrated that the emergence of the ∼70–80 ms response was likely driven mainly by enhanced inhibitory synaptic gain, which could alone account for the observed tACS effects (compare red curves in Figures 9B,E). Excitatory synaptic gain alone (green curve, Figure 9A) tended to move the ∼70–80 ms peak in the opposite direction. Comparison of corresponding spiking patterns of individual cell types for the default parameter set versus the simulation of enhanced total synaptic gain (Figures 9C,D spike histograms, respectively) revealed that while the ∼70–80 ms peak is larger in amplitude in the increased gain condition, there is also less firing in network pyramidal neurons from ∼70 to 80 ms post-stimulus (compare spiking raster plots in yellow boxes in Figures 9C,D). Such decreased firing in layer 5 pyramidal neurons could underlie decreased perception in the post-alpha tACS condition.

## Discussion

### Summary

The intention of this study was to investigate whether brief and intermittent tACS (6 s on/off) at participants’ endogenous alpha frequency could modulate alpha power in the somatosensory system, and whether the hypothesized modulation would causally impact detection of tactile stimuli at perceptual threshold. Replicating our prior MEG results (Jones et al., 2007, 2010), we found that EEG-measured pre-stimulus alpha power (C3 electrode) was higher on non-perceived (“miss”) than perceived (“hit”) trials, and analogous perceptual correlates emerged in early components of the tactile evoked response. We also found that tactile detection performance decreased over time for sham and alpha tACS groups, and there was a significant difference in tactile detection performance between alpha and sham tACS groups during, but not after, tACS. We did not observe evidence of alpha power modulation following tACS. However, we did find that baseline alpha power predicted post- alpha and sham tACS alpha power. In addition, the EEG-measured tactile stimulus evoked response following alpha tACS was marked by the apparent emergence of a prominent peak ∼70–80 ms post-stimulus, which was not discernible in the pre-sham, post-sham or pre-alpha tACS subject averages. Computational neural modeling suggested that a possible interpretation of this ERP effect could be an increase in synaptic gain- inhibitory synaptic gain in particular. These preliminary findings from a small sample (*n* = 6/group) of participants suggest that tACS may affect somatosensory perception via mechanisms related to synaptic plasticity in the absence of lasting measurable changes in spontaneous alpha oscillations. However, this interpretation must be considered with a precautionary note; our study design and experimental set-up had limitations that resulted in the loss of data and a small sample size, and therefore may not have been optimal for measuring lasting oscillatory changes, as discussed further below.

### Lessons Learned From Negative Findings: Lack of Evidence for Lasting Behavioral Effects and Alpha Power Modulation Following Alpha-tACS

Although tactile detection performance differed significantly between sham and alpha tACS groups during stimulation, we did not find evidence to support a lasting behavioral difference between groups during the post-stimulation time period. We also did not observe modulation of EEG-measured alpha power over somatosensory cortex (C3 electrode) following tACS, as originally hypothesized based on prior reports in visual and auditory systems (e.g., Zaehle et al., 2010; Neuling et al., 2012; Helfrich et al., 2014; Vossen et al., 2015). Simultaneous EEG-tACS studies are notoriously difficult, and we experienced complications with data collection. This was due in part to technical difficulties synchronizing our open-source EEG system with proprietary devices in our experimental setup. Although our Open Ephys + EEG system allowed for parameter customization that was crucial to our original experimental design, interfacing with a commercially available tACS device was non-trivial. This represented a significant challenge to data collection, resulting in signal artifact and the loss of EEG data during tACS, as well as the loss of entire datasets. As such, a paramount reason we may not have observed clear evidence of lasting tACS-induced alpha power modulation was our small sample size. Out of 27 recruited subjects, a total of 20 datasets could be used for behavioral analysis, of which only 12 could be used for EEG analysis. Technical challenges also reduced the overall signal to noise ratio of our remaining EEG data. These observations suggest that future studies should implement stimulation and recording devices with established compatibility in order to ensure reliable acquisition of clean EEG data concurrent with electrical stimulation, which may mitigate data loss and enable a larger sample size. Nevertheless, despite this loss of data, observed alterations of the post-tACS evoked response provide evidence that tACS may impact circuit dynamics (Figure 8).

Variation in study design is likely a large source of variability in results across tACS studies (Vossen et al., 2015). While most studies reporting alpha power modulation employ a prolonged period of electrical stimulation (e.g., ∼10–21 min; Zaehle et al., 2010; Neuling et al., 2012, 2013; Helfrich et al., 2014), the current study design employed a brief and intermittent tACS stimulation paradigm (6 s on/off for ∼15 min; Figure 1E) in an attempt to observe immediate effects on EEG-measured cortical dynamics. However, post-processing revealed that EEG data collected during the tACS time block were contaminated with substantial electrical artifact, and therefore could not be analyzed. Analysis of the post-tACS time block revealed that our stimulation protocol (6 s on/off; Figure 1C) may not have been sufficient to induce observable prolonged alpha power modulation (Figure 6). Vossen et al. (2015) utilized a similar design, with either 3 or 8 s trains of alpha tACS lasting 7,200 cycles (∼11–15 min). Prolonged modulation of alpha power was observed following 8 s, but not 3 s, stimulation intervals. Intermittent stimulation at 1 s intervals also failed to produce measurable aftereffects of tACS (Strüber et al., 2015). Although prior studies have not explicitly tested the effects of *6-s* intervals of tACS (used in the present study) compared to longer or shorter trains, we cannot rule out the possibility that the temporal length of stimulation in our experiment was insufficient to induce a similar effect. This suggests that stimulation intervals of at least 8 s in length may be necessary to engage mechanisms of synaptic plasticity that can lead to lasting changes in oscillatory dynamics.

Alternatively, it is possible that in certain behavioral paradigms, such as the present tactile detection task, endogenous alpha rhythms do not always emerge as continuous sustained oscillations, but rather as brief periods of increased power, i.e., “events” or “bursts” (e.g., see Jones et al., 2009; Jones, 2016; Sherman et al., 2016; Shin et al., 2017). For example, prior work from our group demonstrated that pre-stimulus beta power observed in MEG signals source-localized to SI also predicts tactile detection (Jones et al., 2010), and that this beta activity occurs as transient events in un-averaged trials, as opposed to a sustained oscillation (Jones, 2016; Sherman et al., 2016; Shin et al., 2017). Moreover, both the rate and timing of such pre-stimulus beta events drives the association between power and detection consistently across species, modalities and brain states (Shin et al., 2017). For example, tactile stimuli preceded within 200 ms by a single beta event were less likely to be perceived. This suggests that the timing of individual intermittent intervals of electrical stimulation, in relation to presentation of the tactile stimulus, may be more important than the length of time that continuous electrical stimulation is applied. Other studies have shown that alpha oscillations can also emerge transiently (e.g., Jones et al., 2009). As such, NIBS strategies that consider more transient temporal dynamics of endogenous alpha rhythms may be more effective at modulating alpha power and/or perception than those that aim to induce or enhance continuous oscillations (e.g., alpha tACS).

Several studies have demonstrated that modulation of rhythmic activity is brain state-dependent (e.g., Alagapan et al., 2016), and it has been specifically suggested that low endogenous alpha activity may be a necessary precondition for modulation of alpha power using NIBS (Lefebvre et al., 2017). Although we did not control for pre-tACS alpha power, we found that baseline alpha power was linearly related to the change in alpha power from pre- to post-tACS time periods (Figure 7). Therefore, we cannot rule out the possibility that we did not have enough participants with low baseline alpha power to observe a significant effect of stimulation on alpha oscillations. However, we did observe the same linear relationship between pre-tACS alpha power and the pre- to post-tACS percent change in power for both sham and alpha tACS groups. This suggests that the relationship between pre-tACS alpha power and post-tACS change in alpha power is independent of stimulation condition.

Another important consideration that has recently spurred merited debate in the field of neuromodulation is the suggestion that stimulation intensities commonly employed in tACS and tDCS studies (including this study, in which 1 mA tACS was applied at the scalp) are of insufficient strength to induce the magnitude of neural spiking or subthreshold currents that would be required for a true entrainment effect (Vöröslakos et al., 2018). While this debate is out of scope of the present study, a recent review pointed out that these conclusions are “based on manipulation of firing rates, irrespective of ongoing activity,” while tACS is a sub-threshold stimulation technique that is thought to subtly shift *state-specific* endogenous neuronal firing probabilities and coherence (Vosskuhl et al., 2018). While it is possible that the electrical stimulation strength utilized in this study was of insufficient magnitude to modulate alpha power, the stimulation parameters used here are in line with other studies reporting task-specific alpha tACS-induced perceptual effects (e.g., Vossen et al., 2015).

The lack of evidence for a lasting between-groups behavioral effect was likely due in part to our study design; our task was designed with dynamic modulation of tactile stimulus intensity, intended to maintain tactile detection rates close to threshold (50% detection). However, stimulus intensity did not change significantly from pre- to post-alpha or sham tACS (Figure 5). Although tactile stimulus strength was tuned to individual subjects’ perceptual thresholds at the beginning of the experiment, in practice, detection rates were variable throughout the experiment, ranging between ∼ 20–70% detection (Figure 4). This variability allowed for measurable behavioral differences between groups during tACS, but the limited dynamic range may have weakened the likelihood of observing a lasting effect. Despite the limited range of behavioral performance, the findings that performance differed between groups during tACS (Figure 4B), and that only the alpha tACS group exhibited a statistically significant difference in performance between the pre- and post-tACS time blocks (Figure 4A), suggest an interesting behavioral effect and reflects the robustness of these findings.

### Potential Mechanisms Underlying tACS Induced Changes in the Tactile-Evoked Response

Alpha generators across visual, auditory and somatosensory systems may differ with respect to circuit connectivity, neuronal populations, location of maximal current induction, etc., and are therefore likely to be differentially susceptible to effects of electrical stimulation. For this reason, stimulation effects consistent with perceptual modulation across sensory systems may genuinely possess different underlying mechanisms. Therefore, a wide range of neurobiological factors representing alternatives to alpha entrainment should be considered (e.g., see Radman et al., 2009; Stagg et al., 2009; Rahman et al., 2013; Reato et al., 2013; Pelletier and Cicchetti, 2015).

Computational neural modeling presents a unique opportunity to test specific mechanistic hypotheses regarding tACS effects at the circuit-level, in order to delineate potential mechanisms of interest for experiments in humans and animal models. Prior studies have used this approach to propose that tACS may affect alpha power and thus perception via a mechanism of stochastic resonance, resulting in neural entrainment (Ali et al., 2013; Herrmann et al., 2016; Lefebvre et al., 2017). Others have provided evidence that synaptic plasticity could increase alpha power independently of entrainment (Zaehle et al., 2010; Polanía et al., 2012; Vossen et al., 2015; Lafon et al., 2017). However, these mechanistic processes are not necessarily mutually exclusive.

Though we did not observe post-tACS alpha power modulation suggested to be indicative of lasting changes in synaptic plasticity (Zaehle et al., 2010; Polanía et al., 2012; Vossen et al., 2015; Lafon et al., 2017), we did observe a difference in pre- and post-tACS ERP amplitudes near ∼70–80 ms. Based on prior modeling work (Jones et al., 2007, 2009), this finding suggests our short interval stimulation protocol (6 s on/off; Figure 1C) may still have had a residual effect on synaptic dynamics. To directly test this possibility, we employed a computational neural model developed by our group specifically to interpret the circuit mechanisms of EEG/MEG signals (Figure 9A; see “Materials and Methods”). The model has previously been applied to study the origin of somatosensory oscillations and tactile evoked responses, as studied here (Jones et al., 2007, 2009; Ziegler et al., 2010; Sherman et al., 2016). Furthermore, our group has recently developed the model into the free, open-source GUI-driven software, Human Neocortical Neurosolver (HNN^2^; Neymotin et al., 2018). We applied HNN to test the specific hypothesis that changes in synaptic gain could reproduce the enhanced ∼70–80 ms peak observed in the post-alpha tACS evoked response (Figure 8). We found that doubling the value of synaptic gain in our model simulation reproduced the enhanced ∼70–80 ms peak observed in our post-tACS EEG data, and appeared to be driven mainly by enhanced inhibitory synaptic gain (Figures 9B,E). We also observed a decrease in firing of layer 5 pyramidal neurons (L5 PNs) in our model simulation from ∼70 to 80 ms post-stimulus (Figures 9C,D). Since these neurons provide the relay of downstream information out of SI, decreased firing in this population may underlie inhibited perception in the post alpha-tACS period, where we observed that behavioral performance was low, and the majority of trials were non-perceived (Figure 4).

Our computational modeling results also suggest that the changes in synaptic plasticity we observed may be preferentially mediated by changes in inhibitory synaptic conductance. In support of this, it has been suggested that electrical stimulation preferentially activates inhibitory networks at low intensities (≤0.4 mA) before switching to excitation at intensities of at least 1 mA (Moliadze et al., 2012). In consideration of recent evidence that ∼75% of scalp-applied currents may be attenuated by soft tissue and skull (Vöröslakos et al., 2018), it is highly likely that our stimulation protocol (1 mA at the scalp) was attenuated to an effective value below 0.4 mA in neocortex, and therefore may have preferentially enhanced inhibitory mechanisms. The notion that the enhanced ∼70–80 ms post-tACS peak may be related to inhibitory mechanisms and decreased perception was somewhat surprising at first, because a larger 70 ms peak (M70) was associated with a greater probability of perception and increased L5 PN firing in our prior report (Jones et al., 2007). However, in that prior study, the larger 70 ms peak was created by increasing the strength of the ∼70 ms “feedback” input (i.e., distal drive), whereas in the current study, the enhanced 70 ms peak emerged from changes in local synaptic plasticity while the parameters of the “feedback” drive remained fixed. The simulations in the current study also suggest that alpha tACS may enhance inhibitory synaptic gain close to the soma of L5 PNs, which increases the downward current flow induced by the ∼70 ms “feedback” drive, and thus creates a larger peak at 70 ms. Enhanced inhibitory synaptic gain at the L5 PN somas coincidentally inhibits PN spiking. While not exhaustive of all possible mechanisms of change induced by tACS, this interpretation of our data exemplifies how the computational modeling tool, HNN, can be useful to test specific hypotheses about circuit generators of macroscale human EEG data.

### Future Directions to Modulate Alpha Power and/or Perception

tACS can be a useful tool for non-invasively probing complex cortical circuits and related behaviors, however, careful attention to experimental design and stimulation implementation is crucial. While we observed that tACS can impact behavioral performance and EEG correlates of perception without observably modulating alpha power (as discussed above), the primary lesson learned in this study was that certain aspects of our experimental design contributed to uncertainty in our conclusions about the impact of tACS on cortical oscillations. There are several other NIBS techniques and alternative experimental design parameters besides those outlined above that may allow for more effective manipulation of the somatosensory alpha rhythm (e.g., Thut et al., 2011). In particular, transcranial magnetic stimulation (TMS) has better temporal and spatial resolution than tACS/tDCS, and can deliver sub- or supra-threshold stimulation (i.e., can drive spiking directly). Novel technical developments regarding stimulation montages and protocols are also likely to substantially improve experimental outcomes (e.g., Vöröslakos et al., 2018). Nevertheless, our findings reinforce the importance of further studies, particularly those including computational neural modeling, to discover and test alternative mechanistic hypotheses regarding NIBS effects. Ultimately, while novel/alternative methods and tools may aid in unraveling the neural mechanisms that give rise to perceptual behavior, and thus reveal optimal neuromodulation strategies, stringent practices in experimental design are necessary for deduction of accurate and concrete conclusions.

## Ethics Statement

This study was carried out in accordance with the recommendations of the federal human subjects regulations (45 CFR 46, AKA: the “Common Rule”). All subjects gave written informed consent in accordance with the Declaration of Helsinki. The protocol was approved by the Brown University Institutional Review Board (FWA 00004460).

## Data Availability Statement

The raw data supporting the conclusions of this manuscript will be made available by the authors, without undue reservation, to any qualified researcher.

## Author Contributions

CB, PB, UA, JS, NP, BG, CM, and SJ contributed conception and design of the study. DS, PB, CB, UA, and JS contributed to data collection. DS performed the data and statistical analysis. DS, CB, CM, and SJ wrote the manuscript.

## Conflict of Interest Statement

The authors declare that the research was conducted in the absence of any commercial or financial relationships that could be construed as a potential conflict of interest.

## Footnotes

1 https://hnn.brown.edu/index.php/tutorials

2 http://hnn.brown.edu

## Supplementary Material

FIGURE S1 Full HNN Model Simulation of the Tactile Evoked Response from 0 to 170 ms. **(A)** The HNN model simulation of the SI threshold-level tactile evoked response was tuned to source localized MEG data from a prior study (Jones et al., 2007), and provided as a default simulation with the HNN software. The SI tactile evoked response is reproduced by a sequence of excitatory synaptic drive through proximal and distal projections, consisting of proximal (“feedforward”) input at ∼25 ms, followed by distal (“feedback”) input at ∼70 ms, and finally by a re-emergent proximal input at ∼135 ms. Histograms of the spiking activity providing these inputs is shown at the top of the figure. This sequence of exogenous input is supported by laminar recordings in animals (e.g., Cauller and Kulics, 1991; see also prior studies Jones et al., 2007, 2009). **(B)** Layer-specific net current dipole activity demonstrates that current flow within long, large spatially aligned layer 5 pyramidal neurons accounts for most of the current dipole signal. **(C)** Simulated spiking activity of individual excitatory (pyramidal; white and blue) and inhibitory (basket; green and red) cells in distinct neocortical layers in response to proximal and distal excitatory synaptic drive. Histograms of spike patterns providing the proximal and distal inputs are shown at the top of the panel in red and green, respectively. This simulation contained 100 pyramidal neurons and 30 inhibitory neurons per layer. A scaling factor of 3000 was multiplied by the model output to match the amplitude of the recorded signal, providing an estimate that ∼600,000 cells contribute to the recorded response.

## References

[B1] Alagapan S. Schmidt S. L. Lefebvre J. Hadar E. Shin H. W. Frohlich F. (2016). Modulation of cortical oscillations by low-frequency direct cortical stimulation is state-dependent. *PLoS Biol.* 14:e1002424. 10.1371/journal.pbio.1002424 27023427 PMC4811434

[B2] Ali M. M. Sellers K. K. Frohlich F. (2013). Transcranial alternating current stimulation modulates large-scale cortical network activity by network resonance. *J. Neurosci.* 33 11262–11275. 10.1523/JNEUROSCI.5867-12.2013 23825429 PMC6618612

[B3] Antal A. Boros K. Poreisz C. Chaieb L. Terney D. Paulus W. (2008). Comparatively weak after-effects of transcranial alternating current stimulation (tACS) on cortical excitability in humans. *Brain Stimul.* 1 97–105. 10.1016/j.brs.2007.10.001 20633376

[B4] Berger H. (1969). On the electroencephalogram of man. *Electroencephalogr. Clin. Neurophysiol.* 28:37. 4188918

[B5] Bingham E. Hyvärinen A. (2000). A fast fixed-point algorithm for independent component analysis of complex valued signals. *Int. J. Neural. Syst.* 10 1–8. 10.1142/S0129065700000028 10798706

[B6] Black C. Voigts J. Agrawal U. Ladow M. Santoyo J. Moore C. (2017). Open Ephys electroencephalography (Open Ephys + EEG): a modular, low-cost, open-source solution to human neural recording. *J. Neural. Eng.* 14:035002. 10.1088/1741-2552/aa651f 28266930 PMC7249234

[B7] Brainard D. H. (1997). The psychophysics toolbox. *Spat. Vis.* 10 433–436. 10.1163/156856897X00357 9176952

[B8] Cauller L. J. Kulics A. T. (1991). The neural basis of the behaviorally relevant N1 component of the somatosensory-evoked potential in SI cortex of awake monkeys: evidence that backward cortical projections signal conscious touch sensation. *Exp. Brain Res.* 84 607–619. 10.1007/BF00230973 1864331

[B9] Dai H. (1995). On measuring psychometric functions: a comparison of the constant-stimulus and adaptive up–down methods. *J. Acoust. Soc. Am.* 98 3135–3139. 10.1121/1.413802 8550938

[B10] Delorme A. Makeig S. (2004). EEGLAB: an open source toolbox for analysis of single-trial EEG dynamics including independent component analysis. *J. Neurosci. Methods* 134 9–21. 10.1016/j.jneumeth.2003.10.009 15102499

[B11] Druschky K. Kaltenhäuser M. Hummel C. Druschky A. Huk W. J. Neundörfer B. (2003). Somatosensory evoked magnetic fields following passive movement compared with tactile stimulation of the index finger. *Exp. Brain Res.* 148 186–195. 10.1007/s00221-002-1293-4 12520406

[B12] Forss N. Salmelin R. Hari R. (1994). Comparison of somatosensory evoked fields to airpuff and electric stimuli. *Electroencephalogr. Clin. Neurophysiol.* 92 510–517. 10.1016/0168-5597(94)90135-X 7527769

[B13] Frey J. N. Mainy N. Lachaux J.-P. Muller N. Bertrand O. Weisz N. (2014). Selective modulation of auditory cortical alpha activity in an audiovisual spatial attention task. *J. Neurosci.* 34 6634–6639. 10.1523/JNEUROSCI.4813-13.2014 24806688 PMC6608137

[B14] Gundlach C. Müller M. M. Nierhaus T. Villringer A. Sehm B. (2017). Phasic modulation of human somatosensory perception by transcranially applied oscillating currents. *Brain Stimul.* 9 712–719. 10.1016/j.brs.2016.04.014 27237962

[B15] Hämäläinen M. Hari R. Ilmoniemi R. J. Knuutila J. Lounasmaa O. V. (1993). Magnetoencephalography theory, instrumentation, and applications to noninvasive studies of the working human brain. *Rev. Mod. Phys.* 65 413–497. 10.1103/RevModPhys.65.413

[B16] Helfrich R. F. Schneider T. R. Rach S. Trautmann-Lengsfeld S. A. Engel A. K. Herrmann C. S. (2014). Entrainment of brain oscillations by transcranial alternating current stimulation. *Curr. Biol.* 24 333–339. 10.1016/j.cub.2013.12.041 24461998

[B17] Herrmann C. S. Murray M. M. Ionta S. Hutt A. Lefebvre J. (2016). Shaping intrinsic neural oscillations with periodic stimulation. *J. Neurosci.* 36 5328–5337. 10.1523/JNEUROSCI.0236-16.2016 27170129 PMC6601804

[B18] Hoechstetter K. Rupp A. Stanèák A. Meinck H. M. Stippich C. Berg P. (2001). Interaction of tactile input in the human primary and secondary somatosensory cortex - A magnetoencephalographic study. *Neuroimage* 14 759–767. 10.1006/nimg.2001.0855 11506548

[B19] Jensen O. Mazaheri A. (2010). Shaping functional architecture by oscillatory alpha activity: gating by inhibition. *Front. Hum. Neurosci.* 4:186. 10.3389/fnhum.2010.00186 21119777 PMC2990626

[B20] Jones S. R. (2016). When brain rhythms aren’t ‘rhythmic’: implication for their mechanisms and meaning. *Curr. Opin. Neurobiol.* 40 72–80. 10.1016/j.conb.2016.06.010 27400290 PMC5056821

[B21] Jones S. R. Kerr C. E. Wan Q. Pritchett D. L. Hämäläinen M. Moore C. I. (2010). Cued spatial attention drives functionally relevant modulation of the mu rhythm in primary somatosensory cortex. *J. Neurosci.* 30 13760–13765. 10.1523/JNEUROSCI.2969-10.2010 20943916 PMC2970512

[B22] Jones S. R. Pritchett D. L. Sikora M. A. Steven M. Hämäläinen M. Moore C. I. (2009). Quantitative analysis and biophysically realistic neural modeling of the MEG Mu Rhythm: rhythmogenesis and modulation of sensory-evoked responses. *J. Neurophysiol.* 102 3554–3572. 10.1152/jn.00535.2009 19812290 PMC2804421

[B23] Jones S. R. Pritchett D. L. Stufflebeam S. M. Hämäläinen M. Moore C. I. (2007). Neural correlates of tactile detection: a combined magnetoencephalography and biophysically based computational modeling study. *J. Neurosci.* 27 10751–10764. 10.1523/JNEUROSCI.0482-07.2007 17913909 PMC2867095

[B24] Kleiner M. Pelli D. Ingling A. Murray R. Broussard C. (2007). What’s new in psychtoolbox-3. *Perception* 36 1–16.

[B25] Klimesch W. Sauseng P. Hanslmayr S. (2007). EEG alpha oscillations: the inhibition-timing hypothesis. *Brain Res. Rev.* 53 63–88. 10.1016/j.brainresrev.2006.06.003 16887192

[B26] Kronberg G. Bridi M. Abel T. Bikson M. Parra L. C. (2017). Direct current stimulation modulates LTP and LTD: activity dependence and dendritic effects. *Brain Stimul.* 10 51–58. 10.1016/j.brs.2016.10.001 28104085 PMC5260488

[B27] Kulics A. T. (1982). Cortical neural evoked correlates of somatosensory stimulus detection in the rhesus monkey. *Electroencephalogr. Clin. Neurophysiol.* 53 78–93. 10.1016/0013-4694(82)90108-0 6173203

[B28] Kulics A. T. Cauller L. J. (1986). Cerebral cortical somatosensory evoked responses, multiple unit activity and current source-densities: their interrelationships and significance to somatic sensation as revealed by stimulation of the awake monkey’s hand. *Exp. Brain Res.* 62 46–60. 10.1007/BF00237402 3956637

[B29] Lafon B. Rahman A. Bikson M. Parra L. C. (2017). Direct current stimulation alters neuronal input/output function. *Brain Stimul.* 10 36–45. 10.1016/j.brs.2016.08.014 27717601 PMC5774009

[B30] Lee S. Jones S. R. (2013). Distinguishing mechanisms of gamma frequency oscillations in human current source signals using a computational model of a laminar neocortical network. *Front. Hum. Neurosci.* 7:869. 10.3389/fnhum.2013.00869 24385958 PMC3866567

[B31] Leek M. R. (2001). Adaptive procedures in psychophysical research. *Percept. Psychophys.* 63 1279–1292. 10.3758/BF03194543 11800457

[B32] Lefebvre J. Hutt A. Frohlich F. (2017). Stochastic resonance mediates the state-dependent effect of periodic stimulation on cortical alpha oscillations. *Elife* 6 1–21. 10.7554/eLife.32054 29280733 PMC5832422

[B33] Lipton M. L. (2006). Ipsilateral hand input to area 3b revealed by converging hemodynamic and electrophysiological analyses in macaque monkeys. *J. Neurosci.* 26 180–185. 10.1523/JNEUROSCI.1073-05.2006 16399685 PMC4465455

[B34] Moliadze V. Atalay D. Antal A. Paulus W. (2012). Close to threshold transcranial electrical stimulation preferentially activates inhibitory networks before switching to excitation with higher intensities. *Brain Stimul.* 5 505–511. 10.1016/j.brs.2011.11.004 22445135

[B35] Murakami S. Okada Y. (2006). Contributions of principal neocortical neurons to magnetoencephalography and electroencephalography signals. *J. Physiol.* 575 925–936. 10.1113/jphysiol.2006.105379 16613883 PMC1995687

[B36] Neuling T. Rach S. Herrmann C. S. (2013). Orchestrating neuronal networks: sustained after-effects of transcranial alternating current stimulation depend upon brain states. *Front. Hum. Neurosci.* 7:161. 10.3389/fnhum.2013.00161 23641206 PMC3639376

[B37] Neuling T. Rach S. Wagner S. Wolters C. H. Herrmann C. S. (2012). Good vibrations: oscillatory phase shapes perception. *Neuroimage* 63 771–778. 10.1016/j.neuroimage.2012.07.024 22836177

[B38] Neymotin S. A. Daniels D. S. Peled N. McDougal R. A. Carnevale N. T. Moore C. I. (2018). *Human Neocortical Neurosolver (HNN)*. 10.5281/zenodo.1446517 PMC7018509 31967544

[B39] Okada Y. C. Wu J. Kyuhou S. (1997). Genesis of MEG signals in a mammalian CNS structure. *Electroencephalogr. Clin. Neurophysiol.* 103 474–485. 10.1016/S0013-4694(97)00043-6 9368492

[B40] Pelletier S. J. Cicchetti F. (2015). Cellular and molecular mechanisms of action of transcranial direct current stimulation: evidence from in vitro and in vivo models. *Int. J. Neuropsychopharmacol.* 18 1–13. 10.1093/ijnp/pyu047 25522391 PMC4368894

[B41] Pelli D. G. (1997). The video toolbox software for visual psychophysics: transforming numbers into movies. *Spat. Vis.* 10 437–442. 10.1163/156856897X00366 9176953

[B42] Polanía R. Nitsche M. A. Korman C. Batsikadze G. Paulus W. (2012). The importance of timing in segregated theta phase-coupling for cognitive performance. *Curr. Biol.* 22 1314–1318. 10.1016/j.cub.2012.05.021 22683259

[B43] Radman T. Ramos R. L. Brumberg J. C. Bikson M. (2009). Role of cortical cell type and morphology in subthreshold and suprathreshold uniform electric field stimulation in vitro. *Brain Stimul.* 2 215–228. 10.1016/j.brs.2009.03.007 20161507 PMC2797131

[B44] Rahman A. Lafon B. Parra L. C. Bikson M. (2017). Direct current stimulation boosts synaptic gain and cooperativity in vitro. *J. Physiol.* 595 3535–3547. 10.1113/JP273005 28436038 PMC5451737

[B45] Rahman A. Reato D. Arlotti M. Gasca F. Datta A. Parra L. C. (2013). Cellular effects of acute direct current stimulation: somatic and synaptic terminal effects. *J. Physiol.* 591 2563–2578. 10.1113/jphysiol.2012.247171 23478132 PMC3678043

[B46] Reato D. Rahman A. Bikson M. Parra L. C. (2013). Effects of weak transcranial alternating current stimulation on brain activity—a review of known mechanisms from animal studies. *Front. Hum. Neurosci.* 7:687. 10.3389/fnhum.2013.00687 24167483 PMC3805939

[B47] Rice D. M. Hagstrom E. C. (1989). Some evidence in support of a relationship between human auditory signal-detection performance and the phase of the alpha cycle. *Percept. Mot. Skills* 69 451–457. 10.2466/pms.1989.69.2.451 2812990

[B48] Romei V. Gross J. Thut G. (2010). On the role of prestimulus alpha rhythms over occipito-parietal areas in visual input regulation: correlation or causation? *J. Neurosci.* 30 8692–8697. 10.1523/JNEUROSCI.0160-10.2010 20573914 PMC6634639

[B49] Sacchet M. D. LaPlante R. A. Wan Q. Pritchett D. L. Lee A. K. C. Hämäläinen M. (2015). Attention drives synchronization of alpha and beta rhythms between right inferior frontal and primary sensory neocortex. *J. Neurosci.* 35 2074–2082. 10.1523/JNEUROSCI.1292-14.2015 25653364 PMC4315835

[B50] Sherman M. A. Lee S. Law R. Haegens S. Thorn C. A. Hämäläinen M. S. (2016). Neural mechanisms of transient neocortical beta rhythms: converging evidence from humans, computational modeling, monkeys, and mice. *Proc. Natl. Acad. Sci. U.S.A.* 113 E4885–E4894. 10.1073/pnas.1604135113 27469163 PMC4995995

[B51] Shin H. Law R. Tsutsui S. Moore C. I. Jones S. R. (2017). The rate of transient beta frequency events predicts behavior across tasks and species. *Elife* 6 17–21. 10.7554/eLife.29086 29106374 PMC5683757

[B52] Siegle J. H. López A. C. Patel Y. A. Abramov K. Ohayon S. Voigts J. (2017). Open Ephys: an open-source, plugin-based platform for multichannel electrophysiology. *J. Neural Eng.* 14:045003. 10.1088/1741-2552/aa5eea 28169219

[B53] Stagg C. J. Best J. G. Stephenson M. C. O’Shea J. Wylezinska M. Kincses Z. T. (2009). Polarity-sensitive modulation of cortical neurotransmitters by transcranial stimulation. *J. Neurosci.* 29 5202–5206. 10.1523/JNEUROSCI.4432-08.2009 19386916 PMC6665468

[B54] Strüber D. Rach S. Neuling T. Herrmann C. S. (2015). On the possible role of stimulation duration for after-effects of transcranial alternating current stimulation. *Front. Cell. Neurosci.* 9:311 10.3389/fncel.2015.00311 PMC4530587 26321912

[B55] Thut G. (2006). α-Band electroencephalographic activity over occipital cortex indexes visuospatial attention bias and predicts visual target detection. *J. Neurosci.* 26 9494–9502. 10.1523/JNEUROSCI.0875-06.2006 16971533 PMC6674607

[B56] Thut G. Veniero D. Romei V. Miniussi C. Schyns P. Gross J. (2011). Rhythmic TMS causes local entrainment of natural oscillatory signatures. *Curr. Biol.* 21 1176–1185. 10.1016/j.cub.2011.05.049 21723129 PMC3176892

[B57] van Dijk H. Schoffelen J.-M. Oostenveld R. Jensen O. (2008). Prestimulus oscillatory activity in the alpha band predicts visual discrimination ability. *J. Neurosci.* 28 1816–1823. 10.1523/JNEUROSCI.1853-07.2008 18287498 PMC6671447

[B58] Vöröslakos M. Takeuchi Y. Brinyiczki K. Zombori T. Oliva A. Fernández-Ruiz A. (2018). Direct effects of transcranial electric stimulation on brain circuits in rats and humans. *Nat. Commun.* 9:483. 10.1038/s41467-018-02928-3 29396478 PMC5797140

[B59] Vossen A. Gross J. Thut G. (2015). Alpha power increase after transcranial alternating current stimulation at alpha frequency (α-tACS) reflects plastic changes rather than entrainment. *Brain Stimul.* 8 499–508. 10.1016/j.brs.2014.12.004 25648377 PMC4464304

[B60] Vosskuhl J. Strüber D. Herrmann C. S. (2018). Non-invasive brain stimulation: a paradigm shift in understanding brain oscillations. *Front. Hum. Neurosci.* 12:211. 10.3389/fnhum.2018.00211 29887799 PMC5980979

[B61] Wan Q. Kerr C. Pritchett D. Hämäläinen M. Moore C. Jones S. (2011). Dynamics of dynamics within a single data acquisition session: variation in neocortical alpha oscillations in human MEG. *PLoS One* 6:e24941. 10.1371/journal.pone.0024941 21966388 PMC3178572

[B62] Worden M. S. Foxe J. J. Wang N. Simpson G. V. (2000). Anticipatory biasing of visuospatial attention indexed by retinotopically specific alpha-band electroencephalography increases over occipital cortex. *J. Neurosci.* 20:RC63. 10.1523/JNEUROSCI.20-06-j0002.2000 PMC6772495 10704517

[B63] Zaehle T. Rach S. Herrmann C. S. (2010). Transcranial alternating current stimulation enhances individual alpha activity in human EEG. *PLoS One* 5:e13766. 10.1371/journal.pone.0013766 21072168 PMC2967471

[B64] Zhang Y. Ding M. (2010). Detection of a weak somatosensory stimulus: role of the prestimulus mu rhythm and its top-down modulation. *J. Cogn. Neurosci.* 22 307–322. 10.1162/jocn.2009.21247 19400673

[B65] Ziegler D. A. Pritchett D. L. Hosseini-Varnamkhasti P. Corkin S. Hämäläinen M. Moore C. I. (2010). Transformations in oscillatory activity and evoked responses in primary somatosensory cortex in middle age: a combined computational neural modeling and MEG study. *Neuroimage* 52 897–912. 10.1016/j.neuroimage.2010.02.004 20149881 PMC2894272

