# Supplementary figures and images for: A Prospective Study of the Impact of Transcranial Alternating Current Stimulation on EEG Correlates of Somatosensory Perception
Source: Front Psychol. 2018 Nov 20;9:2117. doi: 10.3389/fpsyg.2018.02117 (PMC6255923; doi:10.3389/fpsyg.2018.02117)

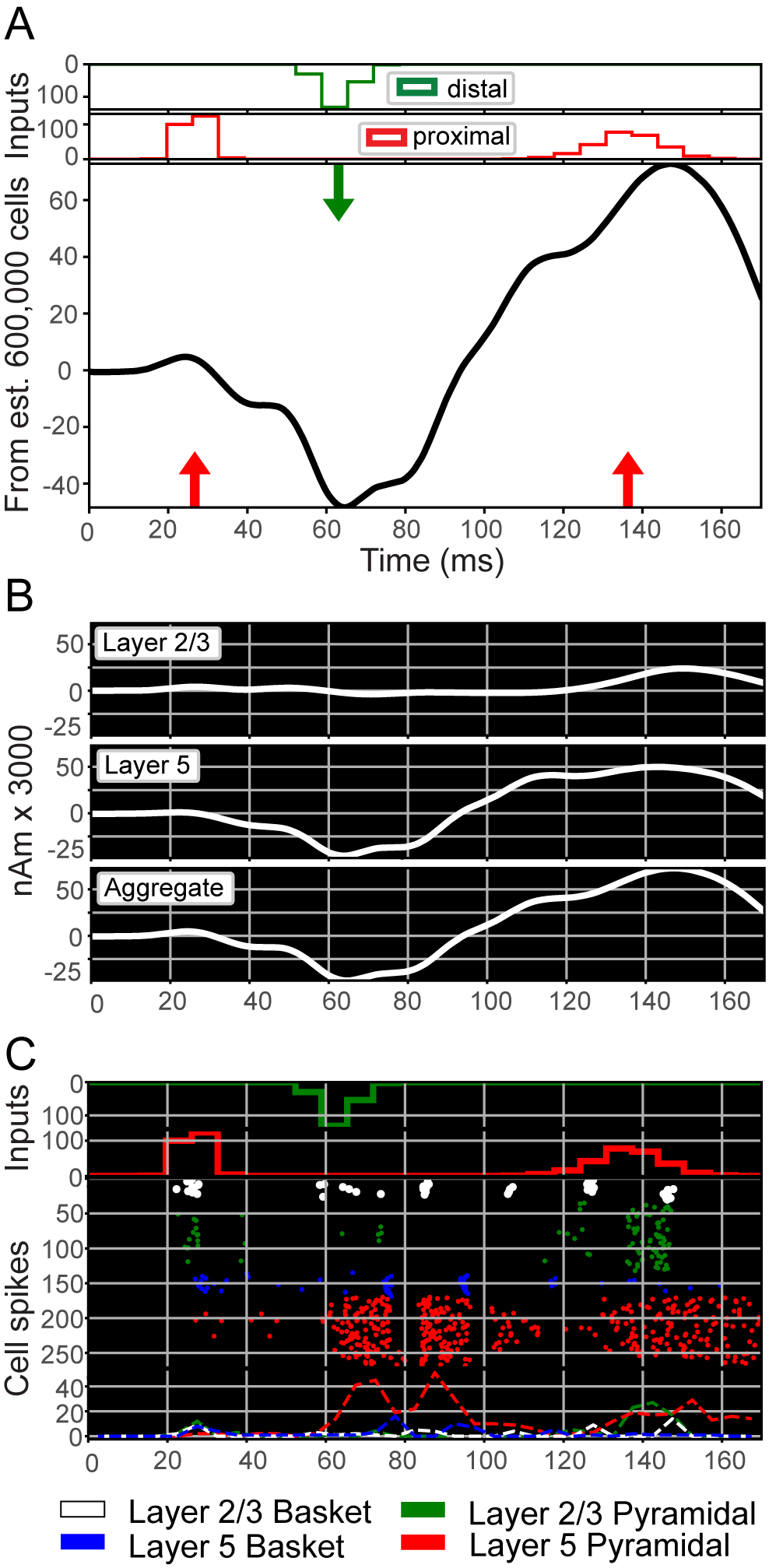

Supplement: FIGURE S1 — Full HNN Model Simulation of the Tactile Evoked Response from 0 to 170 ms. (A) The HNN model simulation of the SI threshold-level tactile evoked response was tuned to source localized MEG data from a prior study (Jones et al., 2007), and provided as a default simulation with the HNN software. The SI tactile evoked response is reproduced by a sequence of excitatory synaptic drive through proximal and distal projections, consisting of proximal (“feedforward”) input at ∼25 ms, followed by distal (“feedback”) input at ∼70 ms, and finally by a re-emergent proximal input at ∼135 ms. Histograms of the spiking activity providing these inputs is shown at the top of the figure. This sequence of exogenous input is supported by laminar recordings in animals (e.g., Cauller and Kulics, 1991; see also prior studies Jones et al., 2007, 2009). (B) Layer-specific net current dipole activity demonstrates that current flow within long, large spatially aligned layer 5 pyramidal neurons accounts for most of the current dipole signal. (C) Simulated spiking activity of individual excitatory (pyramidal; white and blue) and inhibitory (basket; green and red) cells in distinct neocortical layers in response to proximal and distal excitatory synaptic drive. Histograms of spike patterns providing the proximal and distal inputs are shown at the top of the panel in red and green, respectively. This simulation contained 100 pyramidal neurons and 30 inhibitory neurons per layer. A scaling factor of 3000 was multiplied by the model output to match the amplitude of the recorded signal, providing an estimate that ∼600,000 cells contribute to the recorded response. [file Image_1.TIF]
